# Sampling Site Matters When Counting Lymphocyte Subpopulations

**DOI:** 10.1371/journal.pone.0041405

**Published:** 2012-07-25

**Authors:** Benson Ogunjimi, Dieter Peeters, Niel Hens, Ronald Malfait, Viggo Van Tendeloo, Pierre Van Damme, Philippe Beutels, Evelien Smits

**Affiliations:** 1 Centre for Health Economics Research and Modeling Infectious Diseases, Vaccine and Infectious Disease Institute, University of Antwerp, Antwerp, Belgium; 2 Translational Cancer Research Unit, Oncology Center, GZA Hospitals Sint-Augustinus, Antwerp, Belgium; 3 Department of Medical Oncology, University of Antwerp and Antwerp University Hospital, Antwerp, Belgium; 4 Interuniversity Institute for Biostatistics and Statistical Bioinformatics, Hasselt University, Hasselt, Belgium; 5 Department of Laboratory Medicine, Antwerp University Hospital, Antwerp, Belgium; 6 Laboratory of Experimental Hematology, Vaccine and Infectious Disease Institute, University of Antwerp, Antwerp, Belgium; 7 Center for Cell Therapy and Regenerative Medicine, Antwerp University Hospital, Antwerp, Belgium; 8 Centre for the Evaluation of Vaccination, Vaccine and Infectious Disease Institute, University of Antwerp, Antwerp, Belgium; 9 School of Public Health and Community Medicine, University of New South Wales, Sydney, Australia; University of Freiburg, Germany

## Abstract

Clinical and scientific work routinely relies on antecubital venipunctures for hematological, immunological or other analyses on blood. This study tested the hypothesis that antecubital veins can be considered to be a good proxy for other sampling sites. Using a hematocytometer and a flow cytometer, we analyzed the cell counts from samples coming from the radial artery, the dorsal hand veins and the antecubital veins from 18 volunteers. Most surprisingly, we identified the greatest difference not to exist between arterial and venous circulation, but between the distal (radial artery & dorsal hand veins) and proximal (antecubital veins) sampling sites. Naïve T cells had a higher cell count distally compared to proximally and the reverse was true for effector memory T cells. Despite these differences there were high correlations between the different sampling sites, which partially supports our initial hypothesis. Our findings are crucial for the future design and interpretation of immunological research, and for clinical practice. Furthermore, our results suggest a role for interval lymph nodes in the trafficking of lymphocytes.

## Introduction

The human blood circulation can be divided into an arterial circulation going towards the tissues and a venous circulation coming from the tissues. Blood cells such as red blood cells and leukocytes could differ in count between the two circulations. However, both in clinical practice and scientific studies on hematological and immunological diseases, physicians and researchers routinely rely on venipuncture as the common source of blood collection. Until now, only a few studies (Table S1) have looked at the differences in cell counts between the venous and arterial circulations, but no study has done this for lymphocyte subpopulations [1]–[7]. In this exploratory study we made within-individual analyses in 18 individuals comparing cell counts for different blood cell populations collected via the radial artery, a dorsal hand vein and an antecubital vein.

## Materials and Methods

This study was approved by the local ethical committee (University Hospital Antwerp). Written informed consent was obtained from all study participants.

### Description of Study Population & Blood Collection

Eighteen individuals aged between 23 y–41 y (average 32 y, median 30 y; 6 females) were recruited and blood samples were taken and analyzed on three different days. Potential participants were screened for immunity interfering diseases and/or medication. One participant received yellow fever and typhus vaccinations one week before the blood sampling, one participant was a known asthma patient taking cetirizine and inhaled corticosteroids, one participant had at the moment of sampling healed hand lesions originating from a clinically probable hand-foot-mouth infection that started 7 days earlier and one participant was treated for a basocellular carcinoma 3 years earlier at the arm not used for sampling. Since all analyses were performed within-individuals, we included these four participants and performed an outlier analysis which showed no major effects (cf. infra).

For each individual, blood samples from the different sampling sites were taken from the same arm and with maximum 30 minutes between the first and the last sample. Both heparinized tubes (135 USP. U. Lithium Heparin, Venosafe Terumo, Leuven, Belgium) for flow cytometric analyses and EDTA (K3 EDTA 5,4 mg, BD Vacutainer, BD Diagnostics, Sparks MD, USA) tubes were collected using vacutainer systems at three locations: the radial artery ‘R’ (using a winged infusion set (23 G); 7 ml heparinized tubes), a dorsal hand vein ‘D’ (using a winged infusion set (23 G), 7 ml heparinized tubes) and an antecubital vein ‘E’ (using a straight needle (21 G), 10 ml heparinized tubes, ‘E’ for elbow). We note that for one participant we could not obtain a radial artery sample due to fainting of the participant.

### Blood Processing and Analysis

All samples were freshly processed and analyzed. A hematocytological cell count expressed as the number of cells per ml of whole blood was obtained using an Advia Siemens Hematology System. Peripheral blood mononuclear cells (PBMC) were isolated by Ficoll-Paque Plus gradient separation (Amersham Biosciences, Uppsala, Sweden). Staining with monoclonal antibodies that bind membrane molecules was performed for 30 min at 4°C. Flow cytometric measurements (BDFACS Aria flow cytometer, BD Biosciences) were performed in two tubes per individual and per sampling site. PBMC in tube 1 were stained for CD3 (PE TexasRed, Invitrogen), CD4 (APC-H7, BD Biosciences), CD8 (PerCP-Cy5.5, BD Biosciences), CD45 (Pacific Orange, Invitrogen), CD45RA (FITC, BD Biosciences), CCR7 (PE-Cy7, BD Biosciences), CD62L (APC, BD Biosciences), CLA (PE, Miltenyi Biotec) and CCR4 (V450, BD Biosciences) (see Table S2). In tube 2, PBMC were stained for CD3 (PE TexasRed, Invitrogen), CD4 (APC-H7, BD Biosciences), CD8 (PerCP-Cy5.5, BD Biosciences), CD45 (Pacific Orange, Invitrogen), CD19 (Pacific Blue, Dako), CD56 (PE-Cy7, BD Biosciences), CD25 (APC, BD Biosciences), and CLA (PE, Miltenyi Biotec) before fixation and permeabilisation of the cells according to the manufacturer’s protocol (eBioscience) (see Table S2). Afterwards, the fixed cells were stained for Foxp3 (Alexa Fluor 488, BD Biosciences). Fluorescence minus one controls were used for CCR7 and CD25 in order to discriminate between negative and positive cells. FlowJo software version 9.3.2 (Tree Star Inc., Ashland, OR) was used for data analysis with 200,000 events analyzed per tube. A lymphogate was defined per sample based on forward and side scatter characteristics of the cells (see Figure S1 illustrating the gating strategy). Within the CD45^+^CD3^+^CD4^+^/8^+^ parent types, we further differentiated into lymphocyte subpopulations using CD45RA and the lymph node markers CCR7 and CD62L resulting in 16 different T cell subpopulations which were further typed with skin homing marker CLA. CD45^+^CD3^+^CD4^+^CD25^+^Foxp3^+^ (regulatory T cells), CD45^+^CD3^−^CD19^+^ (B cells) and CD45^+^CD3^−^CD56^+^ (natural killer cells) cells were also typed with CLA (assessed using the median fluorescence intensity). All cell counts <400 were omitted from analyses (routine practice due to Poisson statistics). Furthermore, all cell types with overall average or median counts <400 were omitted as well (6/19 cell types, being all CCR7^+^CD62L^−^ T cells, CD4^+^CCR7^−^CD62L^−^CD45RA^+^ and CD8^+^CCR7^+^CD62L^+^CD45RA^−^ T cells). Table S3 shows the average number of cells per lymphocyte subpopulation. We note that CCR4 was also added to tube 1 as a marker for skin homing. However, due to the increasing literature showing CCR4 to be involved in T cell regulation and marking of Th2 cells, we chose not to interpret results from this marker.

### Data Analysis and Statistics

All hematocytological cell counts were analyzed using the number of cells per volume-unit. In order to correct for a potential effect of hemoconcentration we performed an additional analysis after division by the number of RBC per ml. Flow cytometric counts were analyzed using percentages of CD3^+^ cells for T cells. CD3^−^CD19^+^ and CD3^−^CD56^+^ cells were expressed as a percentage of CD45^+^ cells (T cells were also calculated as a percentage of CD45^+^ cells for the absolute counts). Absolute flow cytometric counts (number of cells per ml of normalized whole blood) were analyzed after multiplication by the factor ‘LYM/RBC’ as obtained by the hematocytological cell counter. We note that this method could lower the power of the analysis due to the addition of an extra element of random error.

An average relative difference ‘ARD’ is calculated for all comparisons. As an example for ‘RD’ and applicable to all cell types (and hematocrit), the ARD  =  ((cell count for dorsal hand vein sampling – cell count for radial artery sampling)/cell count for radial artery sampling) averaged over all individuals. This thus calculates the average relative increase or decrease from sampling site R towards sampling site D for a specific cell count.

The primary aim of our analysis was to identify potential differences in cell counts between the three different sampling sites. Therefore per individual and per cell type three comparisons were made: radial artery vs. dorsal hand veins ‘RD’, radial artery vs. antecubital veins ‘RE’ and dorsal hand veins vs. antecubital veins ‘DE’. Univariate two-sided paired t-tests were performed for all comparisons and in case of non-normality (even after natural logarithm transformation) a Wilcoxon matched-pair signed rank test was performed. Univariate results were defined as significant when P<.05 and with a tendency for significance when P<.10. In addition, a multiple testing analysis was performed using the well-known conservative Bonferroni correction adjusting the upper boundary of significance to P<.05/n with n being the number of simultaneous assessed tests. Furthermore, we used the less conservative Benjamini correction [8], in which the number of false-positive results are controlled by P< m*0.05/n with m the number of univariate significant results. In our setting we recognize three different n values: n_1_ for the hematocytological count, n_2_ for the flow cytometric count without CLA and n_3_ for the flow cytometric count with CLA. We also note that the use of the Bonferroni correction has received much criticism in the past (see for instance reference [9]) and that a common mechanism explaining all univariate significant results can be regarded as an acceptable alternative to the multiple testing corrections. Pearson and Spearman correlations were calculated for all comparisons. Univariate testing was performed using Excel for Mac 14.1.3 and PASW Statistics 18.0.3 for Mac.

Finally, in order to account for correlations between the different lymphocyte subpopulations, we performed a multivariate analysis of variance for the lymphocyte subpopulations using the procedure ‘Proc mixed’ in SAS 9.2 with unstructured covariance matrices for the repeated measurements and between the different lymphocyte subpopulations. Contrast statements determined the univariate differences between the sampling sites as well as an overall multivariate P value for all univariate comparisons with P<.10. Studentized residuals were normally distributed and showed no departures from homoscedascity. The multivariate analysis could not be performed for the absolute cell counts.

Cook’s D was used to identify outliers for the multivariate analysis of the uncorrected lymphocyte subpopulations, but the omission of these outliers did not reduce the statistical significance of our results. An additional outlier analysis (using the ARD) performed for the univariate tests on the raw datasets confirmed this finding for both the lymphocyte subpopulations and the hematocytological counts except for two cell types (added as a note to the corresponding tables). For some cell types the P values improved without having a major effect on the overall analysis and conclusions. There were no outlying participants for the hematocytological count, nor for the lymphocyte subpopulation counts. A univariate outlier analysis was also performed for the median fluorescence intensities of CLA. The results after omission of outliers showed no major differences for the comparisons between the radial artery and the antecubital veins. Examining tube 1 solely identified one outlier; a participant with overall higher CLA median fluorescence intensities at the antecubital veins as compared to the other sampling sites. This participant was not identified as an outlier for the analysis of cell types nor was the participant identified as an outlier for tube 2. Combining the univariate outlying participant counts for tube 1 & tube 2 identified an additional, although much less extreme, outlying participant who also had a few higher CLA median fluorescence intensities in tube 1 at the antecubital veins.

## Results

### Monocyte, Platelet and Basophil Counts Differ between Distal and Proximal Sampling Sites

Counts of different blood cell types and hematocrit were determined using a hemocytometer and the results are presented in Table 1 and Figure S2. The Average Relative Difference between two sampling sites showed a significant increase in platelets and monocytes at the antecubital veins as compared to the dorsal hand veins and the radial artery. An increase in leukocytes and neutrophils from the radial artery towards the antecubital veins was detected as well. We did not note differences for lymphocytes nor for eosinophils. Furthermore, we noted a systematic increase in hematocrit from radial artery over dorsal hand veins towards antecubital veins and an associated increase in red blood cell count (+2.7%) at the venous circulation as compared to the arterial circulation. These results suggest that interstitial plasma retention could lead to increased density of the different blood cell types. Therefore, we also calculated normalized results aiming to correct for this phenomenon as shown in Table 1. Although after normalization the results for platelets and monocytes remained similar, the increase in leukocytes and neutrophils from the radial artery towards the antecubital veins was no longer present. In addition, after normalization the basophil counts were shown to decrease from the dorsal hand veins towards the antecubital veins, however without a difference between arterial and venous circulation.

**Table 1:** Comparisons between the hematocytological counts at different sampling sites.

Raw							Normalized				
Cell type	Sites	ARD	Univariate	T/W	Bonferroni	Benjamini	ARD	Univariate	T/W	Bonferroni	Benjamini
PLT^a^	RE	21.4	0.031*	T	0.0018	0.012	19.0	0.099	T	0.0018	0.0037
PLT^a^	DE	5.7	0.045*	T	0.0018	0.012	5.7	0.06	ln	0.0018	0.0037
MON	RE	9.1	0.011*	W	0.0018	0.012	6.4	0.028*	W	0.0018	0.0037
MON	DE	4.8	0.073	W	0.0018	0.012	4.6	0.094	W	0.0018	0.0037
BAS	DE	−12.6	0.083	W	0.0018	0.012	−13.0	0.025*	W	0.0018	0.0037
NGC	RE	3.1	0.039*	Ln	0.0018	0.012					
LEU	RE	3.9	0.060	T	0.0018	0.012					
RBC	RD	2.7	0.00089*	T	0.0018*	0.012*	NA				
RBC	RE	2.6	0.0034*	T	0.0018	0.012*	NA				
HCT	RD	3.0	0.000053*	T	0.0018*	0.012*	NA				
HCT	RE	4.0	0.000022*	T	0.0018*	0.012*	NA				
HCT	DE	1.3	0.064	T	0.0018	0.012	NA				

Hematocytological counts are shown for all comparisons with P<.10 for both the raw and normalized cell counts. The univariate T/W method shows the P value for the two-sided paired t-test ‘T’ (‘Ln’ when a natural logarithm was applied) or Wilcoxon paired-matched signed rank (‘W’) in case normality didn’t hold. The Bonferroni and Benjamini columns show the adjusted significance levels according to the method applied. Sensitivity for outliers was investigated for the raw cell counts. See Materials & Methods for more information.

Results annotated with ‘*’ are considered significant for the method applied. *Sites* comparison between sampling sites; *ARD* average relative difference; *PLT* platelets; *MON* monocytes; *BAS* basophils; *NGC* neutrophils; *LEU* leukocytes; *RBC* red blood cells; *HCT* hematocrit; *RD* radial artery vs. dorsal hand veins; *RE* radial artery vs. antecubital veins; *DE* dorsal hand veins vs. antecubital veins; *NA* not applicable.

a After omission of outliers the ARD for RE reduced to 3.4% and for DE to 3.7% with significances similar to before.

Pearson and Spearman correlations showed a strong and statistically significant correlation (>0.9) for all comparisons, except for the correlations of the platelet and basophil counts between the radial artery and dorsal hand veins which ranged between 0.70 and 0.85 (statistically significant).

### Naïve T Cell Counts Decrease and Effector Memory T Cell Counts Increase from Distal Towards Proximal Sampling Sites

Flow cytometry was applied to count the different lymphocyte subpopulations. As stated in the Materials & Methods section, we could not analyze the results for CD4^+^/CD8^+^CCR7^+^CD62L^−^ and CD4^+^CCR7^−^CD62L^−^CD45RA^+^ T cells due to low cell counts (Table S3).

The results showed that both CD4^+^ and CD8^+^ CCR7^+^CD62L^+^CD45RA^+^ T cells (naïve T cells) had a significantly lower cell count (averaging −8%) at the antecubital veins compared to both the dorsal hand veins and the radial artery (Table 2 and Figure S3).

**Table 2:** Comparisons between the lymphocyte subpopulation counts at different sampling sites.

Relative counts									Absolute counts				
Lymphocyte subpopulation	Sites	ARD	Univariate	T/W	Mixed	Mixed joint	Bonferroni	Benjamini	ARD	Univariate	T/W	Bonferroni	Benjamini
CD4^+^CCR7^+^CD62L^+^CD45RA^+^	RE	−9.6	0.071	W	0.0092*	0.0011*	0.0012	0.0089	−9.4	0.003*	W	0.0012	0.0051*
CD4^+^CCR7^+^CD62L^+^CD45RA^+^	DE	−10.5	0.014*	W	0.0064*	0.0011*	0.0012	0.0089	−9.8	0.064	W	0.0012	0.0051
CD8^+^CCR7^+^CD62L^+^CD45RA^+^	DE	−4.3		T	0.0067*	0.0011*	0.0012	0.0089	−3.6	0.078	W	0.0012	0.0051
CD4^+^CCR7^−^CD62L^+^CD45RA^+^	DE	−2.3		T	0.075	0.0011*	0.0012	0.0089					
CD4^+^CCR7^−^CD62L^+^CD45RA^-^	DE	3.8	0.087	T	0.037*	0.0011*	0.0012	0.0089				0.0012	0.0051
CD8^+^CCR7^−^CD62L^+^CD45RA^-a^	RE	9.6	0.047*	T	0.059	0.0011*	0.0012	0.0089					
CD8^+^CCR7^−^CD62L^+^CD45RA^-^	DE	8.4	0.0066*	T	0.0011*	0.0011*	0.0012	0.0089*	9.1	0.043*	T	0.0012	0.0051
CD4^+^CCR7^−^CD62L^−^CD45RA^-^	RE	9.1	0.017*	T	0.0023*	0.0011*	0.0012	0.0089	10.0	0.036*	T	0.0012	0.0051
CD4^+^CCR7^−^CD62L^−^CD45RA^-^	DE	9.9	0.000097*	T	0.0001*	0.0011*	0.0012*	0.0089*	10.4	0.00032*	T	0.0012*	0.0051*
CD8^+^CCR7^−^CD62L^−^CD45RA^-^	RE	8.4	0.042*	T	0.035*	0.0011*	0.0012	0.0089					
CD8^+^CCR7^−^CD62L^−^CD45RA^-^	DE	5.5	0.037*	T	0.066	0.0011*	0.0012	0.0089					
CD4^+^CD25^+^foxp3^+^	RD	−4.3	0.062	T			0.0012	0.0089					
CD4^+^CD25^+^foxp3^+b^	RE	−5.1	0.062	W	0.035*	0.0011*	0.0012	0.0089					

Lymphocyte subpopulation counts are shown for all comparisons with P<.10 for both the relative and absolute cell counts. The univariate T/W method shows the P value for the two-sided paired t-test ‘T’ (‘Ln’ when a natural logarithm was applied) or Wilcoxon paired-matched signed rank (‘W’) in case normality didn’t hold. The Bonferroni and Benjamini columns show the adjusted significance levels according to the method applied. Sensitivity for outliers was performed for the relative cell counts. The mixed column shows all comparisons with P<.10 assessed as contrasts within a multivariate analysis for the relative counts for the lymphocyte subpopulations using SAS 9.2. The mixed joint column shows the multivariate P value. See Materials & Methods for more information.

Results annotated with ‘*’ are considered significant for the method applied. *Sites* comparison between sampling sites; *ARD* average relative difference; *PLT* platelets; *NGC* neutrophils; *WBC* white blood cells; *RBC* red blood cells; *HCT* hematocrit; *RD* radial artery vs. dorsal hand veins; *RE* radial artery vs. antecubital veins; *DE* dorsal hand veins vs. antecubital veins.

a After omission of outliers the P value from the paired t-test was 0.093 with ARD 6.7%. The multivariate analysis showed a P value of 0.014.

b After omission of outliers the P value from the paired t-test was 0.11 with ARD 3.3%. The multivariate analysis showed a P value of 0.042.

For both CD4^+^ and CD8^+^ CCR7^−^CD62L^−^CD45RA^−^ T cells (effector memory T cells) we noted an increase between 7% and 24% at the antecubital veins as compared to the radial artery and dorsal hand veins. Similar results were obtained for the CCR7^−^CD62L^+^CD45RA^−^ T cells.

CD4^+^CCR7^+^CD62L^+^CD45RA^−^ (central memory CD4^+^ cells), CD8^+^CCR7^+^CD62L^+^CD45RA^−^ (central memory CD8^+^ cells) and CD8^+^CCR7^−^CD62L^−^CD45RA^+^ (effector CD8^+^ cells) T cells showed no significant differences between sampling sites.

We found no differences between sampling sites for natural killer or B cells. However, we observed a tendency for a decrease in CD4^+^CD25^+^Foxp3^+^ T cells (regulatory T cells) from arterial towards venous circulation.

The lymphocyte subpopulations showed strong and significant correlations (correlation coefficients >0.86) for the relative and absolute cell counts.

### Skin Homing Marker CLA Presence on Effector Memory CD8+ T Cells, B Cells and T Regulatory Cells Increases from the Radial Artery Towards the Antecubital Veins

We found the median fluorescence intensity of CLA showing a marked increase from the radial artery towards the antecubital veins for effector memory CD8^+^ T cells, regulatory T cells and B cells and to a lesser extent for effector CD8^+^ T cells and natural killer cells (Table 3).

**Table 3:** Comparisons between the lymphocyte subpopulation median fluorescence intensities of the skin homing marker CLA at different sampling sites.

Raw							Without outliers		
Lymphocyte subpopulation	Sites	ARD	Univariate	T/W	Bonferroni	Benjamini	ARD	Univariate	T/W
CD4^+^CCR7^+^CD62L^+^CD45RA^−^	RE						3.1	0.048*	T
CD4^+^CCR7^+^CD62L^+^CD45RA^−^	DE						2.8	0.073	T
CD4^+^CCR7^−^CD62L^−^CD45RA^−^	RE	16.0	0.098	Ln	0.0012	0.0077	6.1	0.081	T
CD8^+^CCR7^−^CD62L^−^CD45RA^−^	RE	26.3	0.004*	W	0.0012	0.0077*	6.1	0.0075*	Ln
CD8^+^CCR7^−^CD62L^−^CD45RA^−^	DE	17.6	0.084	W	0.0012	0.0077	1.4	0.6	T
CD8^+^CCR7^−^CD62L^−^CD45RA^+^	RD	8.7	0.035*	T	0.0012	0.0077	4.2	0.11	T
CD8^+^CCR7^−^CD62L^−^CD45RA^+^	RE	35.0	0.053	Ln	0.0012	0.0077	7.5	0.0069*	T
CD4^+^CD25^+^foxp3^+^	RD	27.8	0.061	T	0.0012	0.0077	10.8	0.21	T
CD4^+^CD25^+^foxp3^+^	RE	36.2	0.0011*	W	0.0012*	0.0077*	27.0	0.0018*	T
CD3^−^CD19^+^	RD	4.7	0.017*	T	0.0012	0.0077	NO		
CD3^−^CD19^+^	RE	8.9	0.00026*	T	0.0012*	0.0077*	NO		
CD3^−^CD19^+^	DE						4.1	0.014*	T
CD3^−^CD56^+^	RE	2.6	0.046*	T	0.0012	0.0077	NO		

Lymphocyte subpopulations with comparisons between sampling sites for the CLA median fluorescence intensities with P<.10 are shown for the raw data and in addition for the data after omission of outliers. Comparisons with P>.10 after omission of outliers and with P<.10 for the raw data are also shown. The univariate T/W method shows the P value for the two-sided paired t-test ‘T’ (‘Ln’ when a natural logarithm was applied) or Wilcoxon paired-matched signed rank (‘W’) in case normality didn’t hold. The Bonferroni and Benjamini columns show the adjusted significance levels according to the method applied See Materials & Methods for more information.

Results annotated with ‘*’ are considered significant for the method applied. *Sites* comparison between sampling sites; *ARD* average relative difference; *RD* radial artery vs. dorsal hand veins; *RE* radial artery vs. antecubital veins; *DE* dorsal hand veins vs. antecubital veins; *NO* no outliers.

## Discussion

This study examined potential differences in hematological cell counts between different sampling sites: the radial artery, the dorsal hand veins and the antecubital veins. Importantly, we calculated cell counts both with and without normalization accounting for the observed hemoconcentration in the venous circulation compared to the arterial circulation. Our observation of an increase in red blood cell count and hematocrit is supported by some [4]–[6] but not all published studies [1], [3], [7].

Without normalization we found a significant increase in platelets and monocytes at the antecubital veins as compared to the dorsal hand veins and the radial artery. With normalization these results remained, but showed only a tendency for significance. Other studies – in which no normalization procedures for hemoconcentration were applied – did not find a difference between venous and arterial circulation for platelets [1], [3]. However, one study showed a higher monocyte count in venous samples [5]. Although we cannot offer a solid explanation for our observations, we suggest further differentiation of monocyte and platelet cell populations would be of interest in future studies. A similar increase in total leukocyte count and neutrophils from the radial artery towards the antecubital veins was only observed when no normalization was applied. For the neutrophils, no such differences were observed in other studies [1], [5], [6]. For the leukocytes, two studies found similar results [2], [5] whereas two other studies found no differences [1], [3]. Our results thus suggest that hemoconcentration could be the sole cause for the observed differences between arterial and venous samples. Finally, basophil counts were shown to decrease from the dorsal hand veins towards the antecubital veins only after normalization, but like Blann et al. [1] we found no differences between arterial and venous circulation. Eosinophils and lymphocytes showed no differences between sampling sites. This agrees with one study [1] but not with another one that found the lymphocyte count to be higher in venous samples than in arterial samples [5].

Flow cytometric analysis showed no differences between sampling sites for central memory T cells, effector T cells, natural killer or B cells and only a tendency of regulatory T cells decreasing from the radial artery towards the venous circulation. However, we found that lymph node homing naïve CD4^+^ and CD8^+^ T cells had lower cell counts at the elbow compared to both sampling sites of the hand. In contrast, CD4^+^ and CD8^+^ T cells without lymph node homing marker CCR7 (including effector memory T cells) predominantly showed higher cell counts at the elbow, relative to both sampling sites of the hand. Although we cannot draw firm biological conclusions from these data, one possible explanation for these differences in lymphocyte counts between sampling sites can be provided by the transit of blood through small lymph nodes in the forearm when blood flows from distal to proximal, thereby adding effector memory T cells to the antecubital veins and withholding naïve T cells from the antecubital veins. Although it is hitherto assumed that the forearm has no lymph nodes, recent research on melanoma has shown the existence of so called ‘interval’ or ‘in-transit’ lymph nodes at other locations than the typical sentinel nodes [10]. Micro-anatomic research has also proven the existence of small ‘ganglions’ located nearby veins in the forearm [11].

Clark et al showed large numbers of T cells in the normal skin, mostly effector memory cells, but central memory and regulatory T cells as well [12], thereby suggesting an effect of skin homing for T cells. We found that effector memory CD8^+^ T cells, regulatory T cells and B cells had a higher median occupancy of the skin homing marker CLA at the antecubital veins compared to the radial artery. This might be explained by the fact that interval lymph nodes could add lymphocytes with skin homing properties via efferent venules thereby creating a more efficient trafficking of lymphocytes. Taken together, our results further broaden the base of evidence – albeit indirectly – for the existence of interval lymph nodes and could therefore also be relevant to oncological research. More direct proof of the existence of interval lymph nodes could be obtained by high resolution nuclear imaging.

Importantly, the overall results showed strong correlations between the cell counts at the different sampling sites.

The importance of sampling site on cell count adds to the long list of possible sources of variability in clinical immunological studies such as sample management (e.g. type of anticoagulant [13], timing of sampling (i.e. diurnal variation [14]), sample processing time [13], [15], sample freezing conditions [15], cell apoptosis [16], etc.), assay setup (e.g. overlapping peptide mixes vs. whole proteins [17]) and analysis of data (e.g. absolute vs. relative cell counts [18]). For an extensive review of all clinical immunological trial caveats see Maecker et al [19].

A potential source of analytical bias in our study is the fact that different types of puncture sets and needles with different luminal widths were used for collecting blood samples from different sampling sites. However, by using 21 G needles for the antecubital veins and the combination of smaller, less traumatic 23 G needles with winged infusion sets for the hand veins and the wrist, our study – by design – mimics common clinical practice. Nonetheless, it would be of interest for future studies to also examine the effects of needle diameter, winged infusion sets and vacutainer dimensions on cell counts. Theoretically, increased shear stress on cells caused by larger needle diameters, longer blood flow trajectories or higher pressure differences could lead to a physical (shedding) or physiological (downmodulation, cleavage) reduction in cell surface receptors which in leukocytes has been primarily noted for neutrophils (for an review see [20]). The only investigative study we could identify on this matter showed no effect of needle diameter on the presence of microparticles [21]. Based on these findings and theoretical considerations, a higher shear stress is expected for the sampling technique using the 23 G needle puncture set (including the prolonged blood flow trajectory). Our lymphocyte results, however, do not show an effect of physical shedding (e.g. no differences between sampling techniques for natural killer cells or B cells) nor of physiological downmodulation of attachment molecules such as CCR7 and CD62L by the higher shear stress created by the 23 G needle sets. Moreover, we hypothesize that the shear stress encountered during the short duration within the needle, is negligible compared to the shear stress encountered within small blood vessels.

Finally, we believe that a next step in this line of research should be a better understanding of the representativeness of PMBC for the overall immune response. Such advancement can underpin an overall model encompassing the entire lymphatic system and PBMC population.

### Conclusion

The comparison of blood cell counts between different sampling sites clearly demonstrated the existence of differences for at least several lymphocyte subpopulations (effector memory T cells, naïve T cells, T regulatory cells), possibly due to the trafficking of lymphocytes through interval lymph nodes. This implies that clinical, translational and fundamental studies examining these cells should be designed using fixed sampling sites. Fortunately, despite these differences, the between-sampling sites correlations were such that routine antecubital venipuncture can be used as a proxy for all sampling sites.

## Supporting Information

Figure S1 **Graphical representation of gating strategy for lymphocyte subpopulations.** The gating algorithm is shown for a representative sample. First, a lymphogate is drawn using SSC and FSC criteria. After positive selection for CD45, cells are divided in CD3^+^ and CD3^−^ cell types. The CD3^+^ cells are further gated to identify CD4^+^ or CD8^+^ cells after which lymph node homing CCR7 and CD62L gating is followed by CD45RA detection. Also CD25^+^foxp3^+^ cell typing is performed on CD4^+^ cells. The CD3^−^ cells are further differentiated to either B cells (CD19^+^) or natural killer cells (CD56^+^). As stated in Materials and Methods, the lowest panel is typed using a different sample tube as compared to the two higher panels, but the gating strategy starting from CD45^+^ is similar.
(TIF)

Figure S2 **Graphical representation of paired raw hematocytological counts at different sampling sites for all 18 individuals.** Each color line represents counts from the same individual in all graphs. Univariate P values are shown for all P<.10. *RBC* red blood cells; *HCT* hematocrit; *PLT* platelets; *LEU* leukocytes; *LYM* lymphocytes; *MON* monocytes; *NGC* neutrophils; *EOS* eosinophils; *BAS* basophils; *R* radial artery; *D* dorsal hand veins; *E* elbow (antecubital veins).
(TIF)

Figure S3 **Graphical representation of paired relative cell counts of lymphocyte subpopulations at different sampling sites for all 18 individuals.** Each color line represents counts from the same individual in all graphs. Univariate P values are shown for all P<.10. Relative cell counts are expressed as the percentage of CD3+ cells, except for CD3−CD56+ and CD3−CD19+ which are expressed as the percentage of CD45+ cells. *R* radial artery; *D* dorsal hand veins; *E* elbow (antecubital veins).
(TIF)

Table S1 **Short description of published studies discussing hematocytological counts at different sampling sites.** Supplementary Table S1 presents a short description of the published studies discussing hematocytological counts at different sampling sites.
(DOCX)

Table S2 **Staining strategy of PBMC for flow cytometric measurement.** Supplementary Table S2 presents the staining strategy of PBMC.
(DOCX)

Table S3 **Absolute count & percentage of lymphocyte subpopulations.** Supplementary Table S3 presents the absolute counts and the percentages of the lymphocyte subpopulations.
(DOCX)
