# Supplementary figures and images for: Sampling Site Matters When Counting Lymphocyte Subpopulations
Source: PLoS One. 2012 Jul 25;7(7):e41405. doi: 10.1371/journal.pone.0041405 (PMC3405139; doi:10.1371/journal.pone.0041405)

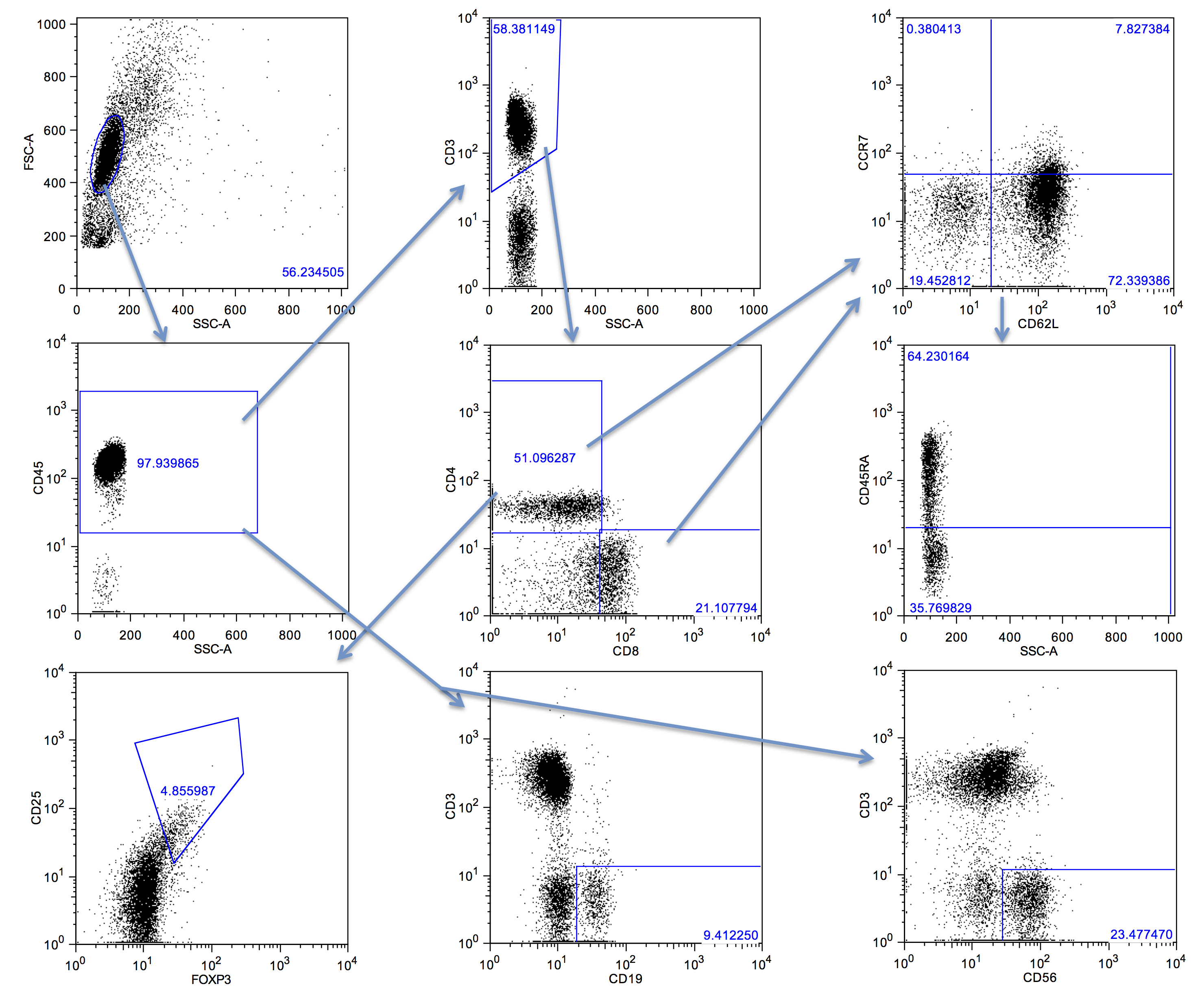

Supplement: Figure S1 — Graphical representation of gating strategy for lymphocyte subpopulations. The gating algorithm is shown for a representative sample. First, a lymphogate is drawn using SSC and FSC criteria. After positive selection for CD45, cells are divided in CD3+ and CD3− cell types. The CD3+ cells are further gated to identify CD4+ or CD8+ cells after which lymph node homing CCR7 and CD62L gating is followed by CD45RA detection. Also CD25+foxp3+ cell typing is performed on CD4+ cells. The CD3− cells are further differentiated to either B cells (CD19+) or natural killer cells (CD56+). As stated in Materials and Methods, the lowest panel is typed using a different sample tube as compared to the two higher panels, but the gating strategy starting from CD45+ is similar. (TIF) [file pone.0041405.s001.tif]

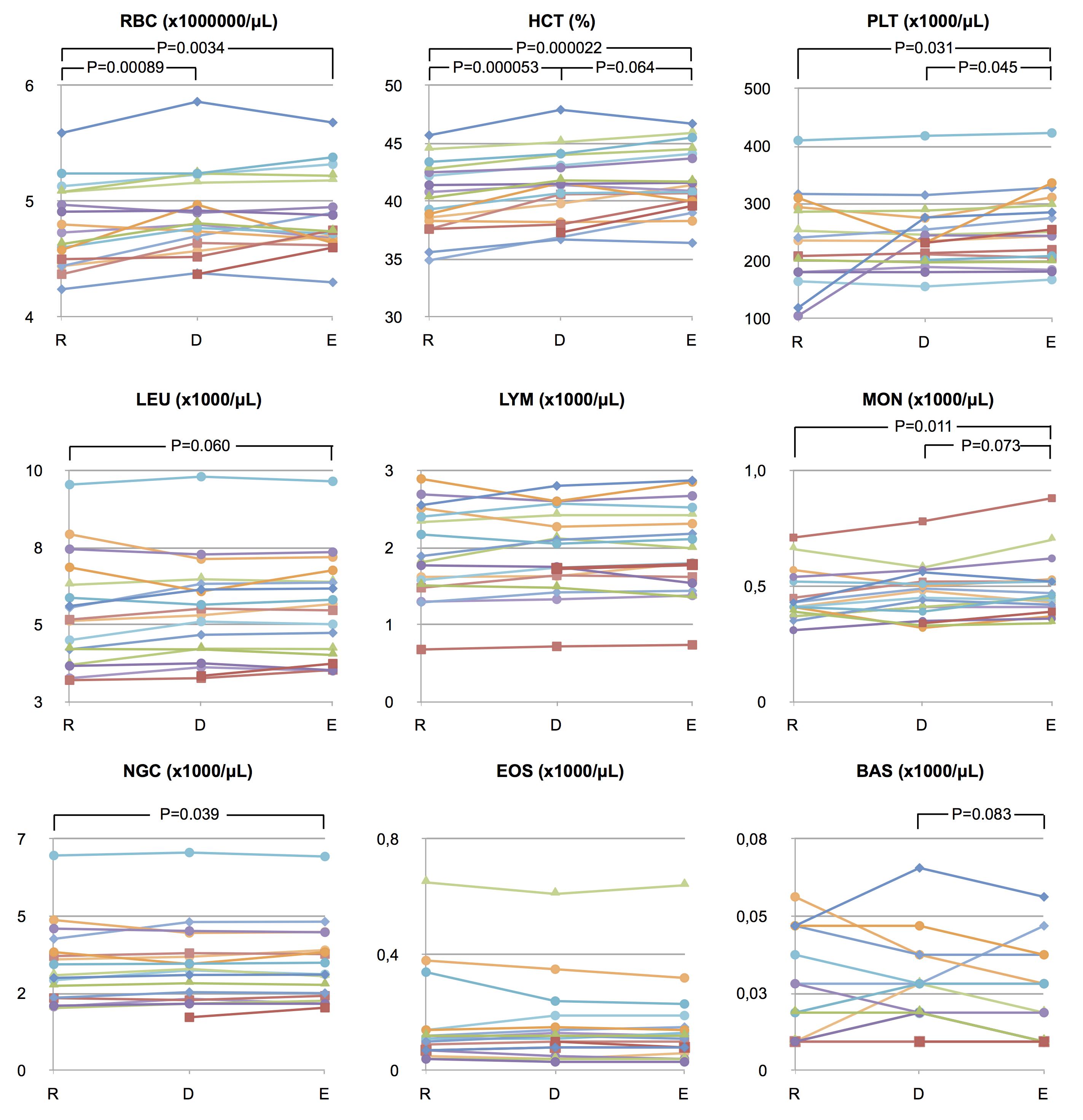

Supplement: Figure S2 — Graphical representation of paired raw hematocytological counts at different sampling sites for all 18 individuals. Each color line represents counts from the same individual in all graphs. Univariate P values are shown for all P<.10. RBC red blood cells; HCT hematocrit; PLT platelets; LEU leukocytes; LYM lymphocytes; MON monocytes; NGC neutrophils; EOS eosinophils; BAS basophils; R radial artery; D dorsal hand veins; E elbow (antecubital veins). (TIF) [file pone.0041405.s002.tif]

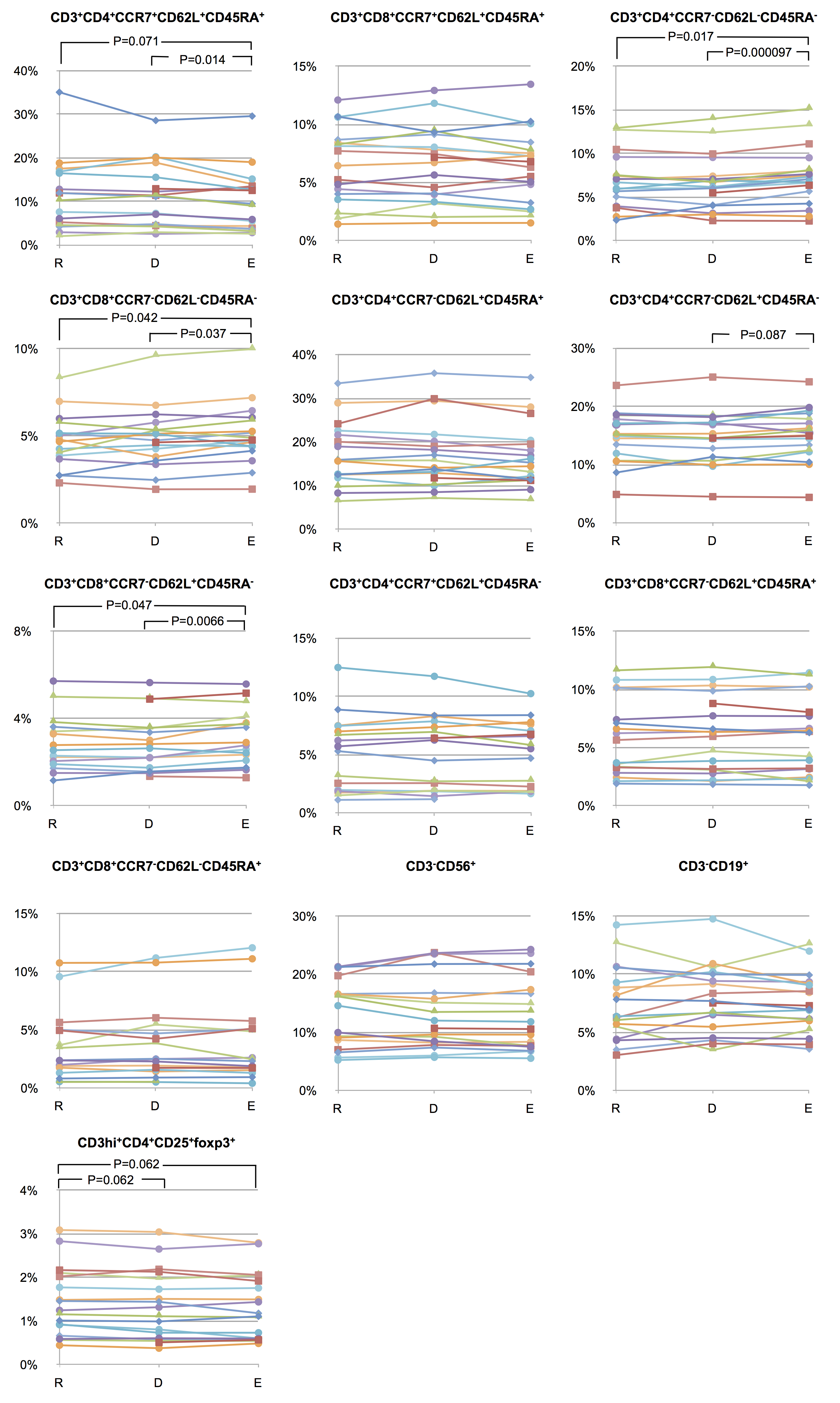

Supplement: Figure S3 — Graphical representation of paired relative cell counts of lymphocyte subpopulations at different sampling sites for all 18 individuals. Each color line represents counts from the same individual in all graphs. Univariate P values are shown for all P<.10. Relative cell counts are expressed as the percentage of CD3+ cells, except for CD3−CD56+ and CD3−CD19+ which are expressed as the percentage of CD45+ cells. R radial artery; D dorsal hand veins; E elbow (antecubital veins). (TIF) [file pone.0041405.s003.tif]
