# Supplementary material for: Sampling Site Matters When Counting Lymphocyte Subpopulations
Source: PLoS One. 2012 Jul 25;7(7):e41405. doi: 10.1371/journal.pone.0041405 (PMC3405139; doi:10.1371/journal.pone.0041405)
Supplement: Table S1 — Short description of published studies discussing hematocytological counts at different sampling sites. Supplementary Table S1 presents a short description of the published studies discussing hematocytological counts at different sampling sites. (DOCX) [file pone.0041405.s004.docx]

Table S1: Short description of published studies discussing hematocytological counts at different sampling sites

| Reference | Year | Study group | n | Sampling sites | Counts |
| --- | --- | --- | --- | --- | --- |
| Christensen et al [[1](#_ENREF_1)] | 1978 | Healthy term neonates or NICU (32-40 weeks gestation) without hematological or bacterial disease | 12 | Capillary (heel puncture), umbilical artery catheter, umbilical vein catheter | LEU, NGC, LYM, HCT |
| Thurlbeck et al [[2](#_ENREF_2)] | 1987 | NICU (24-32 weeks gestation) with RDS and umbilical arterial catheter | 13 (capillary), 21 (dorsal hand vein) | Capillary (heel puncture), umbilical artery catheter, dorsal hand vein | Hb, NGC |
| Blann et al [[3](#_ENREF_3)] | 1996 | Patients with atherosclerosis, connective tissue disease or suspected arteriovenous malformation | 22 | Left antecubital vein, right iliac artery catheter | LEU, NGC, LYM, MON, EOS, BAS, RBC, Hb, PLT |
| Mokken et al [[4](#_ENREF_4)] | 1996 | Patients before coronary artery bypass and/or aortic valve surgery operation | 20 | Radial artery, intravenous catheter in large vein in contralateral arm | HCT |
| Palsgaard-Van Lue et al [[5](#_ENREF_5)] | 2006 | Canines undergoing diverse surgical procedures | 11 | Dorsal pedal artery catheter, jugular vein | LEU, NGC, LYM, MON, RBC, Hb, HCT, PLT |
| Son et al [[6](#_ENREF_6)] | 2010 | Healthy volunteers | 12 | Left radial artery, left median cubital vein | HCT |
| Durila et al [[7](#_ENREF_7)] | 2010 | Severe sepsis patients | 44 | Radial artery catheter, subclavian vein catheter | LEU, RBC, Hb, PLT |

Legend

For each study (shown by reference and year of publication) the study group, the sample size (n), the sampling sites and the type of cell counts are shown.

Footnotes

*NICU* neonatal intensive-care unit; *RDS* respiratory distress syndrome; *LEU* leukocytes; *NGC* neutrophils; *HCT* hematocrit; *Hb* hemoglobin; *LYM* lymphocytes; *MON* monocytes; *EOS* eosinophils; *BAS* basophils; *PLT* platelets

References

1. Christensen, R. D. and Rothstein, G. (1979) Pitfalls in the interpretation of leukocyte counts of newborn infants. Am J Clin Pathol 72, 608-11.

2. Thurlbeck, S. M. and McIntosh, N. (1987) Preterm blood counts vary with sampling site. Arch Dis Child 62, 74-5.

3. Blann, A. D., Adams, R. A., Katai, F., Ashleigh, R., Taberner, D. A. (1996) Haematology and coagulation indices in paired samples of arterial and venous blood from patients with arterial disease. Haemostasis 26, 72-8.

4. Mokken, F. C., van der Waart, F. J., Henny, C. P., Goedhart, P. T., Gelb, A. W. (1996) Differences in peripheral arterial and venous hemorheologic parameters. Ann Hematol 73, 135-7.

5. Palsgaard-Van Lue, A., Jensen, A. L., Strom, H., Kristensen, A. T. (2007) Comparative analysis of haematological, haemostatic, and inflammatory parameters in canine venous and arterial blood samples. Vet J 173, 664-8.

6. Son, K. H., Lim, C. H., Song, E. J., Sun, K., Son, H. S., Lee, S. H. (2010) Inter-species hemorheologic differences in arterial and venous blood. Clin Hemorheol Microcirc 44, 27-33.

7. Durila, M., Kalincik, T., Jurcenko, S., Pelichovska, M., Hadacova, I., Cvachovec, K. (2010) Arteriovenous differences of hematological and coagulation parameters in patients with sepsis. Blood Coagul Fibrinolysis 21, 770-4.
