# Supplementary material for: Sampling Site Matters When Counting Lymphocyte Subpopulations
Source: PLoS One. 2012 Jul 25;7(7):e41405. doi: 10.1371/journal.pone.0041405 (PMC3405139; doi:10.1371/journal.pone.0041405)
Supplement: Table S2 — Staining strategy of PBMC for flow cytometric measurement. Supplementary Table S2 presents the staining strategy of PBMC. (DOCX) [file pone.0041405.s005.docx]

Table S2: Staining strategy of PBMC for flow cytometric measurement

| Specific antibodies | | | | |
| --- | --- | --- | --- | --- |
| Tube 1 | | Tube 2 | | |
| Target | Fluorochrome | Target | Fluorochrome |  |
| CD45RA | FITC | Foxp3 | AlexaFluor 488 |  |
| CLA | PE | CLA | PE |  |
| CD3 | PE-TexasRed | CD3 | PE-TexasRed |  |
| CCR7 | PE-Cy7 | CD56 | PE-Cy7 |  |
| CD8 | PerCP-Cy5.5 | CD8 | PerCP-Cy5.5 |  |
| CCR4 | V450 | CD19 | Pacific Blue |  |
| CD62L | APC | CD25 | APC |  |
| CD45 | Pacific Orange | CD45 | Pacific Orange |  |
| CD4 | APC-H7 | CD4 | APC-H7 |  |

Legend

In order to discriminate different cell populations, PBMC were stained with a cocktail of antibodies that each bind a specific molecule (‘Target’) and are conjugated with a specific fluorochrome (‘Fluorochrome) for multicolor flow cytometric detection.
