# Supplementary material for: Sampling Site Matters When Counting Lymphocyte Subpopulations
Source: PLoS One. 2012 Jul 25;7(7):e41405. doi: 10.1371/journal.pone.0041405 (PMC3405139; doi:10.1371/journal.pone.0041405)
Supplement: Table S3 — Absolute count & percentage of lymphocyte subpopulations. Supplementary Table S3 presents the absolute counts and the percentages of the lymphocyte subpopulations. (DOCX) [file pone.0041405.s006.docx]

Table S3: Absolute count & percentage of lymphocyte subpopulations

| Lymphocyte subpopulation | | | Count (range) | % |
| --- | --- | --- | --- | --- |
| CD4^+^ |  |  | 40392 (11032-68356) |  |
|  | CD4^+^CCR7^+^CD62L^-^ |  | 395 (32-1288) | 1.0 |
|  |  | CD4^+^CCR7^+^CD62L^-^CD45RA^+^* | 34 (3-124) | 8.6 |
|  |  | CD4^+^CCR7^+^CD62L^-^CD45RA^-^* | 361 (29-1201) | 91.4 |
|  | CD4^+^CCR7^+^CD62L^+^ |  | 12025 (1366-34976) | 29.8 |
|  |  | CD4^+^CCR7^+^CD62L^+^CD45RA^+^ | 8167 (921-27912) | 67.9 |
|  |  | CD4^+^CCR7^+^CD62L^+^CD45RA^-^ | 3858 (184-12106) | 32.1 |
|  | CD4^+^CCR7^-^CD62L^+^ |  | 22462 (7646-38650) | 55.6 |
|  |  | CD4^+^CCR7^-^CD62L^+^CD45RA^+^ | 11515 (3508-25894) | 51.3 |
|  |  | CD4^+^CCR7^-^CD62L^+^CD45RA^-^ | 10947 (1175-20973) | 48.7 |
|  | CD4^+^CCR7^-^CD62L^-^ |  | 5510 (1379-14965) | 13.6 |
|  |  | CD4^+^CCR7^-^CD62L^-^CD45RA^+^* | 454 (52-3355) | 8.2 |
|  |  | CD4^+^CCR7^-^CD62L^-^CD45RA^-^ | 5056 (603-11610) | 91.8 |
| CD8^+^ |  |  | 17744 (3535-33494) |  |
|  | CD8^+^CCR7^+^CD62L^-^ |  | 314 (57-831) | 1.8 |
|  |  | CD8^+^CCR7^+^CD62L^-^CD45RA^+^* | 112 (7-488) | 35.7 |
|  |  | CD8^+^CCR7^+^CD62L^-^CD45RA^-^* | 202 (17-691) | 64.3 |
|  | CD8^+^CCR7^+^CD62L^+^ |  | 4815 (1228-10334) | 27.1 |
|  |  | CD8^+^CCR7^+^CD62L^+^CD45RA^+^ | 4555 (1200-9908) | 94.6 |
|  |  | CD8^+^CCR7^+^CD62L^+^CD45RA^-^* | 259 (28-811) | 5.4 |
|  | CD8^+^CCR7^-^CD62L^+^ |  | 6627 (943-14805) | 37.4 |
|  |  | CD8^+^CCR7^-^CD62L^+^CD45RA^+^ | 4466 (828-10240) | 67.4 |
|  |  | CD8^+^CCR7^-^CD62L^+^CD45RA^-^ | 2160 (115-6452) | 32.6 |
|  | CD8^+^CCR7^-^CD62L^-^ |  | 5988 (1215-17910) | 33.8 |
|  |  | CD8^+^CCR7^-^CD62L^-^CD45RA^+^ | 2458 (203-12151) | 41.1 |
|  |  | CD8^+^CCR7^-^CD62L^-^CD45RA^-^ | 3530 (103-6878) | 59.0 |
| CD45^+^ |  |  | 149216 (79079-170936) |  |
|  | CD3^-^CD56^+^ |  | 19857 (6084-38263) | 13.3 |
|  | CD3^-^CD19^+^ |  | 11513 (2960-23074) | 7.7 |
| CD4^+^ |  |  | 61031 (36832-77027) |  |
|  | CD4^+^CD25^+^foxp3^+^ |  | 1402 (465-3563) | 2.3 |

Legend

The flow cytometric counts (originating from 200000 PBMC) are averaged over all individuals and sampling sites per lymphocyte subpopulation. The minimum and maximum range is shown as well. The percentage of a cell type is shown with the parent group as denominator. Cell types annotated with ‘*’ were omitted from further analyses due to low cell counts (See Materials & Methods).
